# Cattle-derived knob paratopes grafted onto peripheral loops of the IgG1 Fc region enable the generation of a novel symmetric bispecific antibody format

**DOI:** 10.3389/fimmu.2023.1238313

**Published:** 2023-10-24

**Authors:** Desislava Yanakieva, Lena Vollmer, Andreas Evers, Vanessa Siegmund, Paul Arras, Lukas Pekar, Achim Doerner, Bernhard Valldorf, Harald Kolmar, Stefan Zielonka, Simon Krah

**Affiliations:** ^1^ Antibody Discovery and Protein Engineering, Merck Healthcare KGaA, Darmstadt, Germany; ^2^ Early Protein Supply and Characterization, Merck Healthcare KGaA, Darmstadt, Germany; ^3^ Targeted mRNA Delivery, Merck KGaA, Darmstadt, Germany; ^4^ Institute for Organic Chemistry and Biochemistry, Technische Universität Darmstadt, Darmstadt, Germany

**Keywords:** AB loop, antibody display, antibody engineering, CH3, cattle antibody, EF loop, ultralong CDR3, yeast surface display

## Abstract

In this work we present a novel symmetric bispecific antibody format based on engraftments of cattle-derived knob paratopes onto peripheral loops of the IgG1 Fc region. For this, knob architectures obtained from bovine ultralong CDR-H3 antibodies were inserted into the AB loop or EF loop of the CH3 domain, enabling the introduction of an artificial binding specificity into an IgG molecule. We demonstrate that inserted knob domains largely retain their binding affinities, resulting into bispecific antibody derivatives versatile for effector cell redirection. Essentially, generated bispecifics demonstrated adequate biophysical properties and were not compromised in their Fc mediated functionalities such as FcRn or FcγRIIIa binding.

## Introduction

During the last decades, monoclonal antibodies (mAbs) and antibody-based products have become one of the major modalities in drug discovery and have shown efficacy in the treatment of various diseases such as cancer, neurological, immunological, and genetic disorders. This became apparent with the approval of the 100th biologic by the FDA in 2021 (1). In the course of time, therapeutic modalities changed from mouse derived monoclonals to fully human antibodies (Abs) and more complex derivates thereof (2–4). Recent clinical success in different fields was made by antibody drug conjugates (ADCs) and bispecific antibodies (bsAbs) (5, 6). Approximately 400 ADCs and 200 bi- and multispecific molecules are currently undergoing clinical investigation (7). Compared to monospecific Abs, bi- and multifunctional Ab derivatives can be harnessed e.g., to redirect T cells to the site of malignant disease or to enhance cancer specificity using paratopes directed against two tumor associated antigens (TAAs) (8–10). Additionally, it is well established that by exploiting bsAbs, major functionalities of natural proteins such as clotting factors or cytokines can be mimicked for disease treatment (11–14).

In terms of generating and engineering bispecific entities, huge progress has been made throughout the years. While initially, bispecifics were made by simply fusing scFvs like beads on a string (15, 16) today many of the formats under clinical investigation are harboring a Fc part for half-life extension or for triggering Fc-mediated effector functions such as antibody-dependent cell-mediated cytotoxicity (ADC), antibody-dependent cellular phagocytosis (ADCP) or complement-dependent cytotoxicity (CDC) (17). However, the generation of Fc-based IgG-like bsAbs is challenging, since two different heavy and two different light chains need to assemble correctly. Different techniques have been described to reach specific heavy chain heterodimerization and correct light chain pairing. Among others, heavy chains can be heterodimerized using knob into holes (KiH) (18), strand exchanged engineered domains (SEEDs) (19) and DEKK mutations (20) while light chains can be forced to pair correctly by electrostatic steering (21) and domain swapping (CrossMab) (22). Moreover, single domain antibodies (23) or common light chain-based (24) molecules can be generated. An alternative approach was described about a decade ago by Wozniak-Knopp and coworkers (25). They have shown that the antibody Fc can serve as a separate binding site. By incorporating mutations into the AB and EF loops of the CH3 domain, antibody fragments binding to HER2 were generated by yeast surface display (YSD). The resulting binders were called Fcab (stands for “Fc antigen binding”). Of note, Fcabs retain all major functions mediated by the Fc proportion of an IgG. Inspired by this work, the current study describes a method to generate bsAbs by incorporating autonomous binding domains into AB or EF loops of a CH3 domain.

It is known since 1990s that a subset of bovine antibodies display a long CDR-H3 region, composed of up to 70 amino acids (26). The V-gene segments of a vast majority of these ultralong CDR3 antibodies belong to IgHV1-7 and preferentially pair with diversity-restricted lambda light chains (V30 segment) (27). Structurally, those CDR-H3s are composed of a stalk and a knob region, with the latter being encoded by the IgHD8-2 gene segment (28). The D-gene knob region harbors four cysteine residues, while 38 codons within the D-gene segment can be mutated to cysteines by just one nucleotide exchange. This results in a large structural diversity of the knob region by different disulfide bond patterns (29). These disulfide bonds rigidify the knob paratope and are crucial for antigen binding (30).

Antigen-specific ultralong CDR-H3 antibodies have been generated against different targets, for instance, viral antigens (31–33) or complement components (34, 35). Moreover, it has been shown that bovine ultralong CDR-H3 antibodies are amenable to humanization to a certain extent (30). Our group recently published a method to isolate ultralong CDRH-3 antibodies after cattle immunization (36). For this, stalk knob regions were specifically amplified and grafted onto a chimeric Fab fragment comprising IgHV1-7 as well as LC V30. Binders against EGFR were subsequently isolated by YSD, and it was shown that a subset of knob architectures can function as autonomous paratopes when expressed as a *N*-terminal Fc fusion, referred to as knobbodies. In a follow-up work, we were able to engineer bispecific antibodies using SEED-technology for heavy chain heterodimerization and ultralong CDRH-3 paratopes directed against EGFR and NKp30, expressed together with a common light chain (V30) (37). These bispecific antibodies led to efficient NK-cell redirection to EGFR expressing tumor cells.

Additionally, as postulated by the group of Macpherson - the close proximity of the *N-* and *C-*termini of bovine knob domains enable transplantation of those small antigen binding regions into different loops of proteins such as rat serum albumin or VHH domains (38). This concept was recently expanded by the same group via inserting serum albumin binding knob architectures into a peripheral loop of framework region III of the VH domain, resulting into half-life extended bispecific Fab fragments (39).

In this study, we engrafted EGFR- and NKp30-directed knobs into either AB or EF loops of an antibody directed against NKp46 (EGFR knobs) or EGFR (NKp30 knobs). EGFR is a receptor tyrosine kinase overexpressed on many solid tumors (40). NKp30 as well as NKp46 are activating receptors belonging to the group of Natural Cytotoxicity Receptors (NCRs) (41). It was previously shown by our group and others that both receptors can be harnessed for efficient NK cell redirection (42–47). We were able to demonstrate that the resulting bivalent bispecific antibodies exhibited good production yields and favorable analytical characteristics in terms of down-stream processing parameters. Moreover, incorporated paratopes were not negatively affected in their affinity when grafted into the antibody Fc region. Resulting bsAbs were able to trigger NK cell-mediated lysis of tumor cells efficiently. Overall, this novel antibody format allows the plug and play generation of stable symmetric IgG-like bsAbs with paratopes originating from cattle immunization, thereby circumventing the need of more laborious synthetic library generation and affinity maturation.

## Materials and methods

### Mammalian cell culturing

Adherent cell lines A431 and MCF-7 were cultivated under standard sterile conditions using DMEM medium + 10% FBS at 37°C in a humidified atmosphere containing 5% CO_2_. Expi293F cells were obtained from Thermo Fischer Scientific and were cultured in a serum-free expression media in a shake flask on an orbital shaker at 110 rpm, 37°C and 8% CO_2_.

### Antibody expression and purification

Constructs were designed as bispecific IgG1-like molecules with CH3 engrafted cattle knobs. DNA constructs were synthesized and subcloned into pTT5 plasmid backbone at GeneArt (Thermo Fisher Scientific) for transient protein expression in Expi293™ cells. To this end, Expi293™ cells were transiently transfected with expression vectors according to the manufacturer’s recommendations (Thermo Fisher Scientific). The antibody-containing supernatants were harvested 5-7 days post transfection and purified via MabSelect antibody purification chromatography resin (GE Healthcare). Antibodies were eluted using 20 mM acetic acid (pH 3.2) and solutions were neutralized using 0.1 volumes 0.5 M sodium phosphate buffer with 1.5 M NaCl (pH 8) to result in a final 1x PBS formulation at pH 6.8. Antibody concentrations were determined via UV-Vis spectrophotometric measurement using the QIAexpert system (Qiagen).

### Size exclusion chromatography

Analytical SEC was performed to assess the purity and aggregation behavior of the antibody derivatives. 7.5 µg protein were injected on TSKgel UP-SW3000 column (4.6 × 300 mm, Tosoh Bioscience LLC) in an Agilent HPLC system under a flow rate of 0.35 ml/min using 50 mM sodium phosphate, 0.4 M NaClO_4_ pH 6.3 as mobile phase. Molecular mass of the proteins was determined using a molecular standard (Bio-Rad).

### Melting point determination

Differential Scanning Fluorometry (nanoDSF) was used for measurement of thermal stability of the generated antibodies using the Prometheus NT.48 (NanoTemper Technologies). The measurement was performed with a linear gradient from 20 to 95°C with temperature increment of 1°C/min and samples run in duplicates using nanoDSF Standard Capillary. The Tonset and midpoints of thermal transitions (Tm) were calculated by the NanoTemper Analysis software PR.ThermControl.

### Biolayer interferometry

To assess binding capacities of the expressed proteins to recombinant antigen, an Octet RED96 system (ForteBio, Pall Life Science) was used with 1000 rpm agitation at 25°C for all experiments.

Initially, binding of the bispecific molecules to both target antigens was verified by loading the proteins at 5 µg/ml in PBS for 3 min on anti-human-Fc (AHC) or anti-human-CH1 (FAB2G) biosensors (Sartorius), followed by a 60 s sensor rinsing step in kinetics buffer (KB; PBS, 0.1% Tween-20 and 1% bovine serum albumin, BSA). Association to recombinant antigens (human EGFR ECD-His_6_ (produced in-house), NKp46-His_6_ (AcroBiosystems), and NKp30-His_6_ (AcroBiosystems) was measured for 180 s using 100 nM protein in KB buffer, followed by dissociation for 120 s in KB.

For kinetics analysis, molecules were loaded as above, followed by a sensor rinsing step in KB for 60 s. Subsequently, association to respective antigens was measured for 300 s in varying concentrations ranging from 3.125 nM to 200 nM for EGFR and 1.6 to 100 nM for NKp46 and NKp30 (diluted in KB) followed by dissociation in KB for 300 s.

The capability of the bispecific constructs to bind simultaneously to their target antigens, was examined by capturing EGFR-His_6_ or NKp30-His_6_ proteins by anti-penta His (HIS1K) biosensors for 3 min at a concentration of 5 µg/ml in PBS, followed by a rinsing step for 60 s in KB buffer. Subsequently, EGFR-loaded biosensors were transferred to antibody solution containing 100 nM of the bispecific anti-NKp46 IgG×a-EGFR knob constructs in KB and incubated for 3 min prior to a final association of the second antigen – 100 nM NKp46-Fc (AcroBiosystems) in KB for 3 min. The bispecific anti-EGFR×a-NKp30 knob constructs were analyzed by association to NKp30-loaded biosensors, followed by association of EGFR-Fc (AcroBiosystems).

To demonstrate intact pH-dependent FcRn binding of the engineered molecules, NKp46-based antibodies (5 µg/ml) were loaded to anti-CH1 (FAB2G) biosensors. For association at pH 6 and pH 7, baseline rinsing was performed in acidic (PBS pH 6 + 0.05% Tween20) or physiological assay buffer (PBS pH 7 + 0.05% Tween20), respectively. Next, 1 µM FcRn diluted either in the acidic or physiological assay buffer were associated for 25 s, followed by a dissociation step under the same pH conditions. Similarly, association at pH 6, followed by a dissociation step in physiological (pH 7) assay buffer was examined.

FcRn binding kinetics of engineered Fc variants was assessed by loading the bispecific antibody constructs to anti-CH1 (FAB2G) biosensors at concentration of 5 µg/ml in PBS. Sensors were rinsed for 60 s in acidic assay buffer (PBS pH 6.0 + 0.05% Tween20) and FcRn-His_6_ (inhouse) was associated at different concentrations (31.25 nM – 1000 nM) for 25 s, followed by a dissociation step at pH 6 for 5 s.

For FcγR binding kinetics, rhFcγRI-ECD-His6 (SinoBiological) or rhFcγRIIIA(176V)-ECD-His6 (R&D Biosystems) were immobilized to anti-penta His (HIS1K) biosensors at a concentration of 5 µg/ml. Association of antibody constructs diluted at ranging concentrations in KB (50 – 3.13 nM for FcγRI and 250 – 3.9 nM for rhFcγRIIIA) was measured for 180 s and 60 s, respectively. Finally, a dissociation step was performed in KB for 600 s or 120 s, respectively.

C1q affinity was measured by loading the antibodies at 5 µg/ml to Protein L (ProtL) biosensors for 180 s. C1q active complex (Abcam) at concentrations in the range 100 – 1.56 nM in KB was associated for 180 s and dissociation in KB was measured for 60 s.

For every BLI experiment appropriate negative controls were included, e.g. an unloaded sensor control to analyze unspecific association of the antigen (K_D_ determination) or an unrelated antigen to validate specificities. Resulting data were fitted and analyzed with ForteBio data analysis software 8.0 using a 1:1 binding model after Savitzky-Golay filtering.

### Flow cytometry

All flow cytometry measurements were performed using iQue3 flow cytometer (Sartorius) in combination with analysis with the corresponding software – IntelliCyt ForeCyt or FlowJo 10.2. C.

Cellular binding properties of NKp46×EGFR-targeting constructs to EGFR-positive A431 and EGFR-negative MCF-7 cells were examined. To this end, 10^5^ cells per well were seeded in a U-bottom 96-well plate and incubated for 1 h at 4°C with antibody constructs serially diluted in PBS + 1% (w/v) BSA (0.06 nM to 1 µM). Subsequently, cells were washed twice with PBS + 1% BSA and incubated with detection antibody solution (1.5 µM of Alexa Fluor^®^ 488 AffiniPure Fab Fragment Goat anti-human IgG (H + L) from Jackson ImmunoResearch) for 30 min at 4°C. Next, cells were washed twice with PBS + 1% BSA and were resuspended in propidium iodide-containing buffer [PBS + 1% BSA + 20 µg/ml PI (Invitrogen)] for dead cell stain. Following controls were included: untreated cells, cells incubated with detection antibody only, and cells treated with cetuximab (positive control) or parental monospecific anti-NKp46 antibody (negative control).

Simultaneous binding of the engineered bispecific constructs to target tumor cells (A431) and soluble NK-activating receptor (NKp30 or NKp46) was assessed. For this, 10^5^ A431 cells/well were seeded and incubated with 100 nM bispecific constructs in presence of 100 nM recombinant his-tagged receptor (NKp30- His_6_ and NKp46-His_6_) for 1 h on ice. Following two washing steps, the cells were incubated with detection antibody mix (10 µg/ml AF488 AffiniPure Fab Fragment Goat anti-human IgG (H + L) (Jackson ImmunoResearch) and 5 µg/ml APC Anti-6x His tag antibody (abcam)) for 30 min at 4°C. Finally, cells were washed twice, resuspended in PI-containing PBS + 1% BSA and analyzed via flow cytometry. Following controls were included: untreated cells, cells incubated with detection antibodies only, and cells treated with monospecific parental IgGs (anti-EGFR and anti-NKp46) as negative controls. Double-positive binding was analyzed using FlowJo 10.2 software.

### Killing assay

Capacities of engineered bispecific molecules to engage and activate NK cells and mediate target cell lysis were evaluated using a fluorescence microscopy-based tumor cell killing assay. Experiments were performed using an Incucyte^®^ Live Cell Analysis System (Sartorius). Human PBMCs were isolated from whole blood samples of healthy donors by density gradient centrifugation, followed by a NK cell isolation via EasySep™ Human NK Cell Isolation Kit (Stemcell Technologies). NK cells were stimulated overnight in AIM V medium (Gibco) supplemented with 100 U/ml recombinant IL-2 (R&D Systems). On the next day, EGFR-positive A431 cells and EGFR-negative ExpiCHO™ cells were stained with CellTracker™ Deep Red Dye (ThermoFisher) according to the manufacturer’s instructions. A total of 2500 stained target cells in 20 μl volume were seeded per well in a 384-well clear bottom microtiter plate (Greiner Bio-One) and allowed to sediment for 3 h at 37°C. Next, 5 μL of antibody solution with varying concentrations ranging from 0.001 pM to 100 nM diluted in medium was added in duplicates to the cells. Subsequently, 12500 NK cells in 20 μL volume were added per well resulting in an effector to target cell ratio of 5:1. Finally, 30 nM SYTOX™ Green Dead Cell Stain (Invitrogen) was dispensed into each well using an automated dispenser (HP). The assay included control wells containing target cells only (untreated cells), effector and target cells without antibody samples (basal killing), effector cells alone, as well as target cells treated with 30 μM staurosporine (Merck Millipore) (maximum killing). Plate incubation (up to 24 h) and online fluorescence imaging was performed using Incucyte^®^ Live Cell Analysis System (Sartorius). For calculation of target cell lysis, the confluence of double-fluorescent cells (green and red) was divided by red fluorescence confluence. Basal killing was subtracted from each sample and all data was normalized to maximum lysis triggered by the therapeutic antibody cetuximab at 10 nM. Four-parameter non-linear regression analysis of GraphPad Prism was used to calculate the IC_50_ killing values.

### Complement-dependent cytotoxicity assay

Cell-based CDC assay was performed using EGFR-positive A431 target cells. 1×10^4^ Target cells were seeded in a 96-well plate and grown overnight at 37°C, 5% CO_2_. On next day, culture medium was removed and samples and baby rabbit complement (Cedarlane) pre-diluted in medium without phenol red were added at final concentrations of 10 nM for antibody samples and 5% v/v for complement. Cells were incubated for 2 h in a humidified atmosphere at 37°C, 5% CO_2_ and then 100 µl/well CellTox Green reagent (Promega) was added. Following shaking for 1 min at 800 rpm and incubation for 15 min at room temperature in the dark, the supernatant was removed, and cells were carefully washed with PBS. Finally, 100 µl/well PBS were added, and fluorescence was measured at 485 nm excitation and 528 nm emission using fluorescence plate reader (Synergy 4, BioTek). Mean fluorescence values from triplicates were normalized to killing by lysis buffer positive control of the CellTox Green kit and plotted as percent of maximum lysis.

### Molecular modeling

To create structural models of the full-length IgG the antibody modeler tool in the molecular modeling software package moe (Molecular Operating Environment 2020.09: Chemical Computing Group Inc.; 2020) was utilized. Homology models of the stalk and NKp30-directed knob regions were generated using moe’s classical homology modeling utility. Structural models of the engrafted knobs into the AB or EF loops were built by adding linkers via moe’s protein builder, followed by a conformational search of the linker via moe’s linker modeler. Finally, energy minimization was performed, treating the linker as flexible and the IgG and knob domains as rigid bodies. Visualization of 3D structures was done with PyMOL (The PyMOL Molecular Graphics System, Version 2.0 Schrödinger, LLC.).

## Results

### Molecular design of symmetric bsAbs with Fc-knob engraftments

**Figure 1:**
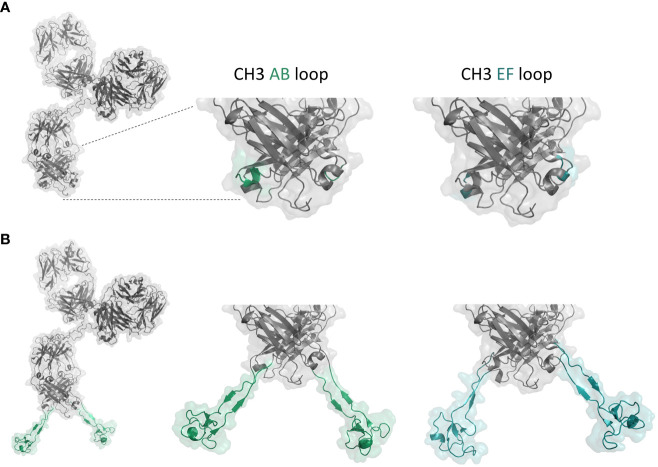
**(A)** Structure model of anti-NKp46 IgG1. Surface-exposed AB and EF loops of the CH3 domain are marked in green and cyan, respectively. **(B)** Structure model of anti-NKp46 IgG with a cattle knob engrafted in the CH3. Anti-EGFR knob H05 engrafted into the AB and EF loops of the CH3 domain are marked in green and cyan, respectively. Figure was generated with PyMOL software version 2.3.0.

In this study, we aimed at developing a novel platform for the efficient generation of symmetric IgG-like bispecific antibodies. To this end, we inserted cattle-derived knob architectures obtained by immunization and YSD (36, 37) into the Fc region of a human IgG1 scaffold, giving rise to antigen-binding Fc regions (**Figure 1**). In an elegant work conducted by Wozniak-Knopp and colleagues it was previously shown that within the IgG1 CH3 domain, the surface-exposed AB as well as EF loops are amenable to randomization or amino acid insertions (25). In this manner, the group was able to create antigen-binding Fc parts, referred to as Fcabs. It was previously demonstrated that cattle-derived knob domains can be engineered to function as autonomous paratopes (36–39). Importantly, these cysteine-rich regions can readily be isolated from bovine immune repertoires after immunization. Moreover, the knob domain itself is rather small in size (3-6 kDa). Hence, it was tempting to speculate that grafting these low molecular weight paratopes into the AB or EF loop of the CH3 domain would not negatively interfere with the natural characteristics of the Fc part, such as stability, antibody folding or Fc-mediated effector functions (**Figure 1B**). In order to demonstrate a general applicability of this approach, we decided to exploit four sequence-diverse knob regions against two different antigens, EGFR (F06 and H05) and NKp30 (D02 and H02) for transplantation (**Supplementary Tables S1**, **S2**). Importantly, knob architectures comprised between four and six cysteine residues which are known to form different disulfide bond patterns. Besides, the length of the four paratopes ranged from 39 to 45 amino acids. For distancing and to enable an adequate spatial orientation of the knob paratope allowing for antigen binding, respective domains were flanked on both termini with a Gly_4_Ser linker. Transplantations into the AB loop were inserted between the PPSR sequence motif (*N-*terminal) and the *C-*terminal NGVS motif, while amino acids EEMTK were replaced by the knob engraftments (**Supplementary Tables S1**, **S2**). In terms of the EF loop, cattle-derived paratopes were transplanted between the *N*-terminal KLTV amino acid motif and the *C*-terminal RWQQ. Three amino acids (DKS) were replaced by the knob engraftment. To get a thorough understanding about bifunctionality, NKp30-specific knob architectures were grafted onto the EGFR-targeting therapeutic antibody cetuximab, while EGFR-directed knob regions were inserted into a NKp46-specific IgG1 antibody (43). At first, the amino acid substitutions L234A and L235A (LALA) were inserted into the Fc region in order to investigate effector redirection capacities of generated symmetric bispecifics independently of FcγR interactons.

### Expression and biophysical characterization of engineered bsAbs

Bispecific molecules with incorporated knob paratopes in the effector silenced IgG1 (L234A, L235A, LALA) CH3 region (IgG×knob bispecifics) as well as corresponding parental IgGs were recombinantly expressed and purified using standard MabSelect affinity chromatography. Expression yields of all generated IgG molecules with engrafted paratopes ranged from the upper double digit to the triple digit milligram per liter scale (75 – 220 mg/l, **Table 1**), generally indicating adequate productivity. In this regard, yields of constructs containing the anti-EGFR knob F06, as well as molecules harboring the anti-NKp30 knob H02 were lower than the corresponding parental IgGs, while constructs with integrated H05 (anti-EGFR) and D02 (anti-NKp30) were comparable. Analytical size exclusion chromatography (SE-HPLC) confirmed low aggregation profiles and high purity of all molecules (>90% target monomer species) (**Supplementary Figure S1**). Additionally, thermal stability of the proteins was assessed by nanoDSF to investigate the impact of bovine knob domain grafting on the biophysical properties of the antibodies. Two midpoints of thermal transitions (Tm) at about 71-72°C and 82-84°C could be observed for both WT antibodies, corresponding to denaturation of CH_2_/Fab domain (Tm1) and CH_3_ domain (Tm2) of the anti-EGFR antibody and to CH_2_ (Tm1) and CH_3_/Fab (Tm2) of the anti-NKp46 antibody (see **Supplementary Figure S2**). For the loop-grafted anti-EGFR antibodies, a decreased Tm2 melting temperature (5-7°C) was observed representing engineered CH_3_ domain unfolding compared to respective WT antibody. No CH_3_ thermal transition could be resolved for anti-NKp46 antibodies due to overlapping transitions from CH_3_ and Fab fragment. For both antibody scaffolds, Tm1 representing the antibody least stable domain (CH_2_ or CH_2_/Fab) was also decreased for all engineered variants, whereas AB loop insertions appeared to be more destabilizing (7-10°C) than EF loop insertions (3-4°C).Overall, transplantation of cattle knobs into peripheral loops of the antibody Fc did not influence protein expression dramatically and was well-tolerated in terms of aggregation properties, while thermal stabilities were moderately affected for EF and AB loop insertions.

**Table 1:** Characterization of IgGs with CH3-engrafted bovine knobs.

Nr.	Construct name	Expression yield (mg/l)	SE-HPLC MabSelect purity (%)	Tm (°C)	Tm1 (°C)	Tm2 (°C)
1	*Anti-NKp46 IgG*	*222.0*	*95.7*	*60.8*	*71.1*	*83.9*
2	Anti-NKp46_AB_F06	110.8	91.3	56.0	62.9	84.6
3	Anti-NKp46_AB_H05	200.3	95.6	57.6	64.1	84.5
4	Anti-NKp46_EF_F06	79.7	98.4	61.8	67.9	84.9
5	Anti-NKp46_EF_H05	224.8	99.3	61.7	68.2	84.9
6	*Anti-EGFR IgG*	*133.9*	*94.6*	*64.2*	*72.0*	*81.6*
7	Anti-EGFR_AB_D02	118.7	92.9	55.9	62.5	74.9
8	Anti-EGFR_AB_H02	83.0	89.4	56.8	63.5	NA
9	Anti-EGFR_EF_D02	98.5	96.1	62.5	68.1	75.4
10	Anti-EGFR_EF_H02	74.5	98.1	60.9	68.0	76.1

Expression yields were determined via BLI of cell culture supernatant prior to affinity chromatography purification. Analytical SEC after MabSelect antibody purification was used to determine the fraction of target monomer species (% purity). Melting point analysis was performed via nanoDSF. All samples were formulated in PBS pH 6.8-7.4.

Italic means reference molecules for comparison.

### Target Binding Affinities

Binding kinetics of the IgG×knob bispecifics to their respective antigens were measured using biolayer interferometry (BLI). For comparison, affinities of the corresponding chimeric cattle IgG molecules and knobbodies (knob-Fc fusions) (36) were determined in the same setting (**Table 2**, **Supplementary Figures S3**, **S4**). In case of the EGFR×NKp30-targeting bispecifics, binding affinities of the anti-EGFR Fab, as well as the anti-NKp30 knob to their respective antigens were comparable to the monospecific anti-EGFR IgG and reference chimeric cattle IgG molecules, respectively. Affinities of NKp46-specific D02 grafts were 3.3 nM for the AB loop insertion and 1.4 nM for the EF loop engraftment, while parental D02 chimeric cattle IgG displayed affinities of 2.8 nM against recombinant human (rh) NKp46. H02 knob grafts had affinities of 1.2 nM (AB loop and EF loop) compared to 2 nM of the parental H02 chimeric cattle IgG. In terms of anti-EGFR knob architectures, the grafted H05 knob retained its double-digit nanomolar binding affinity against (rh) EGFR (37.7 nM for anti-NKp46_AB_H05 *vs.* 30.1 nM for anti-NKp46_EF_H05 *vs.* 27.1 nM for H05 chimeric cattle IgG) regardless of the insertion position. The measured affinity was approximately 2-fold higher in comparison to the H05-Fc fusion knobbody (67 nM). An impact of the knob integration site upon its binding affinity was only unveiled in case of the F06 anti-EGFR knob. In this respect, affinities of F06 transplanted into the EF loop were similar to that of the corresponding parental F06 chimeric cattle IgG (9.7 nM for anti-NKp46_EF_F06 *vs.* 8.9 nM for F06 chimeric cattle IgG) whereas the F06 AB loop graft showed diminished binding of 29.4 nM. In summary, all tested knob insertions largely retained binding against their cognate antigen. Integration site-specific differences in binding kinetics were only observed for the F06 knob paratope.

**Table 2:** Determination of binding affinities of IgG×knob bispecifics to respective target proteins via BLI (**Supplementary Figures S3**, **S4**).

Nr.	Sample	Target 1	K_D_ (nM)	Target 2	K_D_ (nM)
1	Anti-NKp46 IgG	EGFR	x	NKp46	1.0
2	Anti-NKp46_AB_F06	EGFR	29.4	NKp46	2.0
3	Anti-NKp46_AB_H05	EGFR	37.7	NKp46	3.4
4	Anti-NKp46_EF_F06	EGFR	9.7	NKp46	3.0
5	Anti-NKp46_EF_H05	EGFR	30.1	NKp46	3.6
*6*	*F06 chimeric cattle IgG*	*EGFR*	*8.9*		
*7*	*H05 chimeric cattle IgG*	*EGFR*	*27.1*		
*8*	*Knobbody_F06*	*EGFR*	*18.1*		
*9*	*Knobbody_H05*	*EGFR*	*67.0*		
10	Anti-EGFR IgG	EGFR	2.6	NKp30	x
11	Anti-EGFR_AB_D02	EGFR	2.5	NKp30	3.3
12	Anti-EGFR_AB_H02	EGFR	2.8	NKp30	1.2
13	Anti-EGFR_EF_D02	EGFR	2.6	NKp30	1.4
14	Anti-EGFR_EF_H02	EGFR	2.9	NKp30	1.2
*15*	*D02 chimeric cattle IgG*			*NKp30*	*2.8*
*16*	*H02 chimeric cattle IgG*			*NKp30*	*2.0*

Italic means reference molecules for comparison.

**Figure 2:**
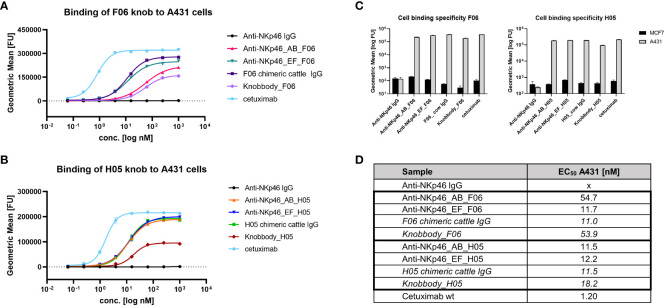
Binding of Fc-engrafted anti-EGFR bovine knobs to EGFR-overexpressing cells. Concentration-dependent binding to A431 cells of F06 **(A)** and H05 **(B)** knobs integrated into anti-NKp46 mAb. **(C)** Cell binding specificity was determined by comparison of binding properties to EGFR-negative MCF-7 cells at 250 nM. **(D)** EC_50_ of A431-binding were calculated using four-parameter non-linear regression analysis of GraphPad Prism 8.0.1 software. Data represents geometric mean of the fluorescence intensity values ± SD of two biological replicates.

Next, binding of NKp46×EGFR-targeting bispecifics to EGFR-overexpressing A431 cells was characterized (**Figure 2**). In accordance with BLI measurements, the anti-NKp46_EF_F06 antibody demonstrated comparable cellular binding as shown for the F06 chimeric cattle IgG, indicating bivalent antigen engagement, whereas anti-NKp46_AB_F06 showed significantly diminished cellular binding capabilities, comparable to the F06 knobbody (~5-fold higher EC_50_; **Figures 2A, D**). H05 knob variants bound comparable to the H05 chimeric cattle IgG, regardless of the loop integration site (**Figures 2B, D**). Additionally, for this paratope, the maximal binding signals of Fc-engrafted H05 paratopes were double as high as the Fc-fused construct (knobbody_H05). Importantly, specific cell binding was detected for all engineered constructs with no unspecific interactions to EGFR-negative MCF-7 cells (**Figure 2C**).

### Simultaneous antigen binding

**Figure 3:**
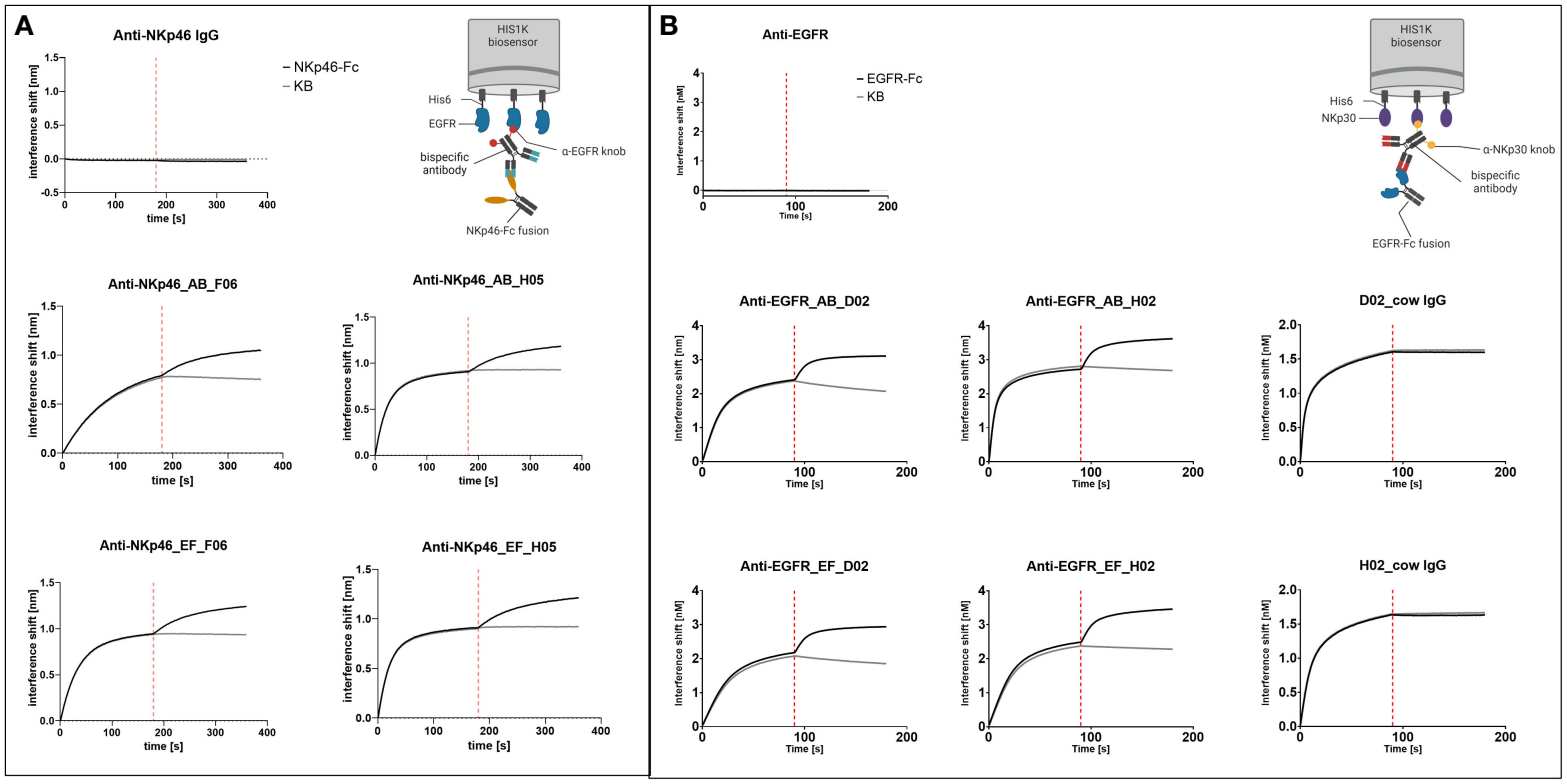
Simultaneous recombinant antigen binding of IgG×knob bispecifics. **(A)** BLI measurements of anti-NKp46 mAbs with Fc-integrated anti-EGFR knobs (F06 and H05). (Rh) EGFR-His_6_ was loaded on HIS1K biosensors, followed by association of the respective constructs at 100 nM. In a subsequent association step, (rh) NKp46-Fc fusion protein was utilized at 100 nM concentration in comparison to a negative control (KB). **(B)** BLI measurements of anti-EGFR mAbs with Fc-integrated anti-NKp30 knobs (D02 and H02). (Rh) NKp30-His_6_ was loaded on HIS1K biosensors, followed by association of the antibody at 100 nM. Next, (rh) EGFR-Fc fusion protein was associated at a concentration of 100 nM in comparison to KB.

Redirection and activation of NK cells requires simultaneous engagement of two antigens. To demonstrate the ability of our engineered molecules to bind both antigens concomitantly, sequential association of the recombinant antigens was detected using BLI (**Figure 3**). In case of the NKp46×EGFR-targeting bispecifics, his-tagged (rh) EGFR was immobilized to anti-his biosensors followed by antibody association (**Figure 3A**). The second association step was conducted using (rh) NKp46-Fc fusion protein resulting in further specific increase in the interference shift for all tested bispecific antibodies in comparison to buffer controls. The EGFR×Nkp30-targeting bispecifics were analyzed in an equivalent manner: His-tagged (rh) NKp30 antigen was immobilized, followed by antibody and subsequent (rh) EGFR-Fc association. Again, all four bispecific constructs demonstrated simultaneous target binding, while no (rh) EGFR-Fc binding could be detected in case of the monospecific anti-NKp30 chimeric cattle IgG molecules (D02_chimeric cattle IgG and H02_chimeric cattle IgG). Furthermore, no unspecific interactions of the monospecific parental anti-NKp46 and anti-EGFR IgGs with immobilized (rh) EGFR and (rh) NKp30 was revealed.

**Figure 4:**
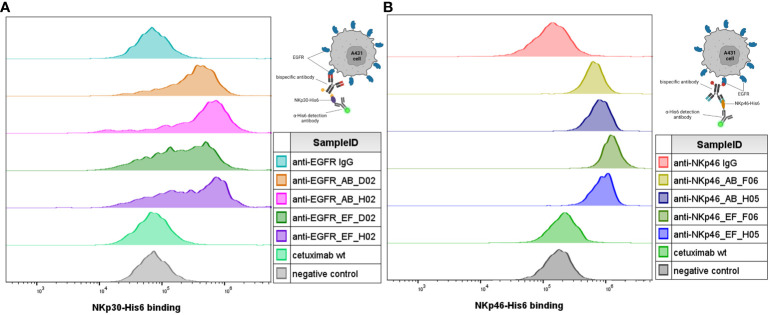
Simultaneous antigen binding of IgG×knob bispecifics on cells. Simultaneous binding to EGFR-overexpressing A431 cells and recombinant NKp30-His_6_ **(A)** and NKp46-His_6_ **(B)** targets was demonstrated by incubation of cells together with 100 nM of the respective antibody construct in presence of 100 nM recombinant target protein (NKp46 for anti-NKp46 IgG-based constructs **(B)** and NKp30 for anti-EGFR-IgG-based constructs **(A)**). Antibody-mediated binding of the recombinant target antigens was detected using fluorescently labeled anti-His_6_ detection. Cells treated with the monospecific parental antibodies were used as a reference and cells incubated with the secondary antibodies only were used as a negative control. Exemplary histograms from two independent experiments.

Next, simultaneous engagement of cell surface-expressed EGFR and (rh) NCRs was verified using flow cytometry (**Figure 4**). EGFR-positive A431 cells were treated with bispecific IgG×knob molecules in presence of soluble recombinant NKp30-His_6_ (**Figure 4A**) or NKp46-His_6_ (**Figure 4B**). Concurrent cell binding and soluble antigen association was detected using anti-His_6_ detection antibody. Increase in the fluorescence signal, indicating specific binding of the His-tagged soluble NK receptors to cell-bound antibody, was detected for all tested bispecific molecules, while monospecific parental IgGs (anti-NKp46 and anti-EGFR IgG) exhibited the same fluorescence intensity as the negative control (anti-His_6_ detection antibody).

### NK cell engagement and targeted cell lysis

**Figure 5:**
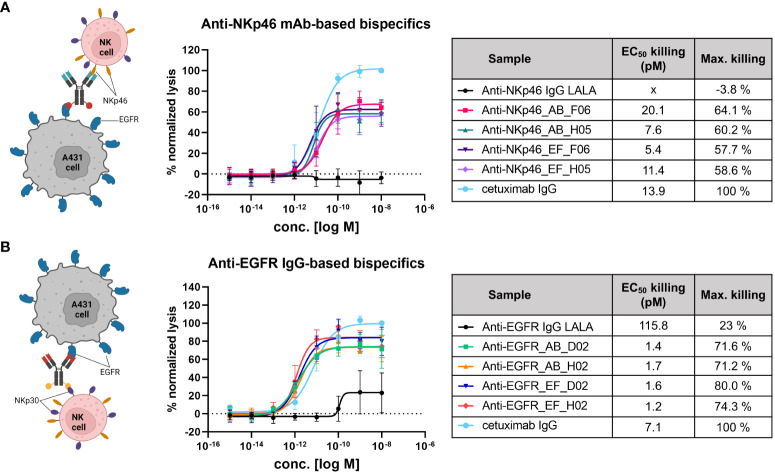
NK cell-mediated tumor cell killing induced by NKp46×EGFR- **(A)** and EGFR×NKp30-targeting bispecifics **(B)**. EGFR-overexpressing A431 cells (T) were incubated with primary NK effector cells (E) at E:T ratio of 5:1 for 4 h in presence of antibody constructs in different concentrations. Tumor cell killing was normalized to maximal cell lysis in presence of 100 nM cetuximab (effector-competent). Killing curves represent data means ± SEM from 4 independent experiments using NK cells from 4 different healthy donors.

Functional activity of IgG×knob bispecific molecules was investigated by target-specific engagement of primary NK cells (via NKp46 or NKp30) using a cytotoxic NK cell-mediated lysis assay (**Figure 5**). Cytotoxic activity was normalized to cetuximab which induces antibody-dependent cell-mediated cytotoxicity (ADCC) through interactions with FcγRIIIa (CD16a) expressed by NK cells. Fc-silenced parental monospecific anti-NKp46 and anti-EGFR IgGs were exploited as negative controls. Since all bispecific constructs are substantially attenuated in terms of FcγR-mediated effector functions via introduction of the LALA mutation, they can induce NK cell mediated tumor lysis primarily by activation and redirection of NK cells due to interactions with NKp30 and NKp46, respectively (**Figure 5** left-hand side). First, the basal killing (target + effector cells without antibody) and the maximal targeted killing (cetuximab) were compared over time, indicating the brightest assay window in the first 6 h of incubation (**Supplementary Figure S5**). Hence, NK cell-mediated lysis was evaluated 4 h post application of test samples for all tested donors. As shown in **Figure 5A**, all NKp46×EGFR-targeting bispecifics mediated similar dose-dependent NK cell engagement and target cell lysis. Potencies were comparable to cetuximab with EC_50_ values in the single to lower double digit picomolar range and efficacies (maximal killing) reaching ~60% of cetuximab-mediated lysis. In case of the EGFR×NKp30-targetig constructs (**Figure 5B**), even higher target cell killing (up to 80% of cetuximab-mediated lysis) and lower EC_50_ (~1.5 pM) values were obtained. While, the Fc-silenced monospecific anti-NKp46 mAb did not mediate any target cell lysis (**Figure 5A** black plot), about 23% of cell killing was detected at higher concentrations of the ADCC-attenuated anti-EGFR IgG (**Figure 5B** black plot). Overall, these experiments demonstrate that transplantation of cattle knob paratopes into peripheral and surface exposed loops of the antibody Fc region is feasible for the generation of functional bsAbs. Furthermore, the presented findings imply no significant influence of the integration site (AB or EF) on effector cell recruitment and immunological synapse formation.

### FcRn and FcγR binding to a cattle knob engrafted antibody

**Figure 6:**
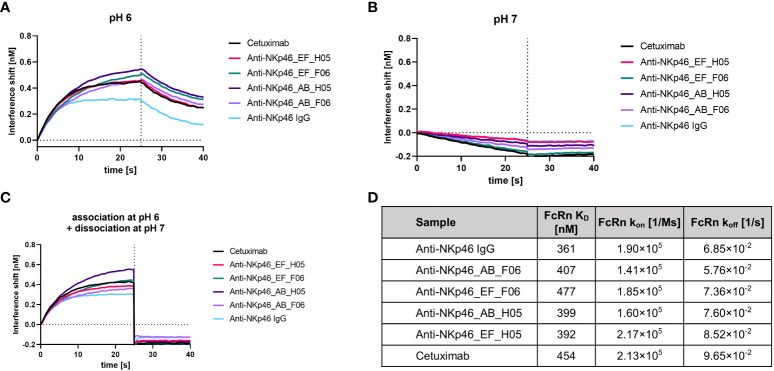
Assessment of FcRn binding after engraftment of bovine-derived knobs into the CH3 domain of an IgG1. **(A)** FcRn (1 µM) binding at pH 6 was investigated after immobilization of the antibodies to FAB2G biosensors. **(B)** No association of FcRn (1 µM) was observed at pH 7 after immobilizing the antibodies to FAB2G biosensors. **(C)** FcRn (1 µM) was associated at pH 6, followed by a dissociation step at pH 7. **(D)** Binding of bispecific molecules to FcRn was compared to the non-modified parental IgG via BLI kinetic analysis (**Supplementary Figure S6**). Overview of calculated FcRn binding affinities and association and dissociation rates at pH 6.

The Fc domain of an antibody is crucial for effector functions like antibody-dependent cell mediated cytotoxicity (ADCC), complement-dependent cytotoxicity (CDC), as well as plasma half-life extension through binding to the neonatal Fc receptor (FcRn). Thus, Fc sequence modifications should be evaluated regarding Fc gamma receptor (FcγR), complement component and FcRn binding properties. As antibody recycling depends on pH-dependent binding of an IgG to FcRn, we investigated the binding of the IgG×knob bispecifics to FcRn under physiological (pH 7) and acidic (pH 6) conditions (**Figures 6A-C**). Association at pH 6 and rapid dissociation at pH 7 comparable to the parental IgG molecule and cetuximab indicate no effects of CH3-grafting on pH-dependent FcRn interactions. Additionally, binding affinities of IgG×knob bispecifics to FcRn were analyzed using kinetic measurements via BLI (**Figure 6D**, **Supplementary Figure S6**). K_D_s of all molecules lay in the same triple-digit nanomolar range (360-480 nM) with no attenuation of FcRn association and dissociation rates in comparison to reference anti-NKp46 and cetuximab antibodies.

For validation of preserved FcγR interaction, we produced anti-NKp46_AB_H05 and anti-NKp46_EF_H05, without LALA mutation. Binding kinetics to the high affinity FcγRI (CD64) and low affinity FcγRIIIa (CD16a) was assessed using BLI (**Supplementary Figures S7A, B**). Specific binding to FcgRI and FcγRIIIa was detected for all NKp46×H05 knob (AB and EF) bispecifics without significant alterations in binding affinities (**Table 3**). Similarly, kinetics of complement component 1 q (C1q) was investigated (**Supplementary Figure S5C**) revealing preserved interaction for AB and EF grafts (**Table 3**). In comparison, the effector silenced anti-NKp46 LALA IgG exhibited as expected strongly reduced association to the tested FcγRs and no interactions with C1q (**Supplementary Figure S7**). Moreover, a cell-based CDC killing assay was performed, in which antibodies were incubated with A431 cells and baby rabbit complement. Significant target cell lysis was detected for cetuximab control only, whereas no significant A431 killing was observed using NKp46×EGFR knob bispecifics. However, when co-incubated with cetuximab, due to bivalent binding of different epitopes, IgG×knob bispecifics exhibited significant tumor cell lysis, although slightly reduced compared to a cetuximab/matuzumab combination (**Supplementary Figure S8**).

**Table 3:** Binding affinities of Fcγ receptors and complement component 1q to effector-competent anti-NKp46×H05 knob bispecific constructs in comparison to the monospecific anti-NKp46 IgG (**Supplementary Figure S7**).

Sample	FcγRI K_D_ (nM)	FcγRIIIA K_D_ (nM)	C1q K_D_ (nM)
Anti-NKp46 eff+	3.8	107	4.1
Anti-NKp46_AB_H05 eff +	2.4	73.5	3.8
Anti-NKp46_EF_H05 eff +	2.4	103	4.1

Taken together, the presented strategy for the generation of symmetrical IgG-like bispecific molecules by grafting of bovine-derived knob paratopes into AB and EF loops of human IgG1 CH3 seems to not impede FcγR interactions (as exemplarily shown for FcγRI and FcγRIIIa), complement component (C1q) and FcRn binding.

## Discussion

BsAbs have shown promising clinical activity in antibody-based therapies since they can mediate novel modes of action unaddressable by conventional monospecific antibodies (48). In previous studies, we investigated whether bovine derived ultralong CDR-H3 paratopes can be efficiently engineered into bsAbs (37). Since ultralong CDR-H3 heavy chains pair typically with a conserved light chain encoded by the VL30 gene-segment, they can be almost considered as natural source of common light chain paratopes. Based on this, cattle derived common light chain bsAbs directed against NKp30 and EGFR were generated that showed promising biophysical properties as well as functionality in terms of effector cell redirection.

In the current study, we sought to investigate whether knob domains can be engrafted into peripheral surface exposed loops of the CH3 domain, referred to as AB and EF loops. This might be beneficial for generating bispecifics, since one specificity can be an available and well characterized antibody paratope, for instance, a binding site derived from an antibody that has been approved for therapy. In addition to that, both paratopes in this novel format allow for bivalent antigen targeting. It has been previously demonstrated that peripheral CH3 loops can be engineered as separate antigen binding sites (25). Synthetic libraries were generated, introducing mutations in AB loop residues 358-362 (EU numbering) and EF loop residues 413-415 as well as 418 and 419. Interestingly, an insertion of 5 amino acids, incorporated at position 415 to increase the size of the newly generated binding site (Fcab), was well tolerated in terms of expression yield and purity. Fcabs directed against HER2 were generated and affinity optimized by yeast display.

In this study, AB loop residues 356-360 or EF loop residues 416-419 were replaced by a G_4_S-knob-G_4_S motif. Utilized knobs comprised four to six cysteine residues and had lengths of 39-51 amino acids. Two knobs with specificities against EGFR (grafted into either AB or EF of an anti-NKp46 antibody) and two knobs with specificities against NKp30 (grafted into either AB or EF of an anti-EGFR antibody) were evaluated in-depth regarding their biophysical properties, binding behavior, and functionality. No tendency for aggregation was observed for all constructs tested, while thermal stabilities were more severely affected for AB than for EF grafts.

Affinities of all CH3-inserted knob domains were compared to the ones of the respective chimeric cattle IgGs (**Table 2**). D02, H02 (directed against NKp30), H05 and F06 (directed against EGFR) CH3 EF loop grafts had similar affinities, while the F06 knob in the AB loop showed slightly reduced binding against EGFR. For both EGFR binding knob grafts, also so called knobbodies were analyzed. Here, the knob domain is *N*-terminally fused to the hinge region of an IgG1 Fc. Interestingly, H05 grafts seem to resemble the affinities of the chimeric cattle IgG while knobbodies showed significantly diminished binding capacities. Consequently, it is tempting to speculate, that the knob architecture needs to be embedded adequately for proper antigen binding. Intriguingly, besides the herein presented findings, it was shown by Macpherson and colleagues, that due to the close proximity of the *N-* and *C-*termini of the knob domain, this paratope can be engrafted onto different proteins (38). Most recently, the same group inserted antigen specific knob regions into a framework III loop of a VH domain, facilitating the creation of bispecific Fab molecules (39). It was demonstrated that the knob domains showed increased affinities when formatted as framework III insertions. The authors speculated that this was due to the VH domain presumably stabilizing both, the *N-* and *C*-termini of the knob domain, similar to the overall architecture in the herein presented study. Interestingly, the AB_F06 (and not the EF loop insertion) variant had a diminished affinity to EGFR comparable to its corresponding knobbody. The same was seen in EC_50_ cellular binding experiments (**Figure 2**) on A431 cells expressing EGFR: all tested bsAbs (except for AB_F06) showed identical cellular binding capacities compared to chimeric cattle IgGs, indicating bivalent antigen binding. Again, knob region F06 engrafted onto the AB loop demonstrated diminished binding capacities, similar to its corresponding knobbody. It is known that within bovine ultralong CDR-H3 antibodies the stalk region has important structural functions (29, 30). Obviously, for engineered knobbody derivatives, the spatial orientation of the knob paratope is quite different from its natural environment. Fascinatingly, besides F06 transplanted into the AB loop of the CH3 domain, it seems that knob-derived paratopes are displayed accordingly to allow for full functionalities in terms of antigen binding.

Because many functionalities of an IgG1 are mediated by the antibody Fc part, it was important to investigate whether those were retained in the IgG×knob bispecifics. Because previous experiments were conducted with effector attenuated antibodies (LALA mutation), H05-harboring molecules were produced with an effector competent Fc. To demonstrate Fc-mediated functionalities, affinities to FcRn, FcγRI, FcγRIIIA and C1q were measured by BLI (**Figure 6**; **Figures S6**, **S7**). All tested bispecifics showed similar binding to those proteins compared to the parental NKp46 IgG.

The ability of the effector-competent NKp46×H05 constructs to induce CDC was further scrutinized in a cell-based CDC assay. Here only cetuximab demonstrated significant lysis of A431 cells. Similar activity of cetuximab has been described by Hsu et al. using A549 cells (49). As it has been previously shown that the combination of antibodies targeting different EGFR epitopes can enhance CDC (50), NKp46×H05 knob bispecifics were co-incubated with cetuximab (non-competing (36)). In this setting, significant killing was observed, likely due to a more efficient C1 recruitment as a result of more pronounced receptor clustering (as shown recently also with a biparatopic anti-HER2 antibody (51)). It has been described that C1 recruitment to IgG-opsonized cells or pathogens is facilitated through a noncovalent hexamerization of the Fc parts of the antigen-bound antibodies resulting in increased apparent avidity of C1q (52). Furthermore, certain mutations in the Fc-Fc hexamer interface reduced the complement activation and CDC while not significantly affecting the C1q binding affinity measured by ELISA (52). Admittedly, the integration of the knob structures into the AB or EF loops may affect Fc-hexamerization and thus reduce their CDC capacity. While induction of CDC has been shown, a head-to-head comparison of EGFR×NKp30 against cetuximab needs to be conducted in the future to finally judge on hexamerization of the novel bispecific format.

In conclusion, we were able to prove the versatility of knob engraftments for the generation of bsAbs, that was likewise demonstrated by Macpherson and colleagues (39). AB and EF loops of an IgG1 have been characterized to be well suited as acceptors for knob domains obtained from cattle immunization. For most of the generated molecules, the grafting of knobs did not significantly impact antibody biophysical characteristics and knob domains were presumably stabilized by the CH3 domain. This was apparent, since most knobs (except for one) bound their antigens with affinities comparable to their native chimeric cattle IgG. Importantly, due to the distance between the antibody Fab and CH3 domain, the affinity of the Fab paratope is not affected and simultaneous binding was mediated by all generated constructs. The herein described format allows the generation of symmetric bivalent (both paratopes) bispecifics in a ‘plug-and-play’ manner without substantial protein engineering and affinity optimizations. To the best of our knowledge, it is the first time that paratopes derived from immunizations (that are consequently highly specific and of high affinity) have been integrated into the Fc portion of an antibody, resulting into antigen-binding Fc regions. Moreover, this approach may also be amenable to the generation of trispecific constructs, exploiting bovine knob domains with different specificities, integrated into an antibody Fc harboring “knob-into-hole” or Duobody mutations, analogous to previously published Fcab-based approaches (53, 54).

## Data availability statement

The original contributions presented in the study are included in the article/**Supplementary Material**. Further inquiries can be directed to the corresponding authors.

## Author contributions

SZ, SK, and DY conceived and designed all experiments. LV and DY performed experiments. DY, SZ, SK, VS, BV, PA, LP, and AD analyzed the data. DY, SZ, and SK wrote the manuscript. AE did molecular modeling studies. HK gave scientific advice. All authors contributed to the article and approved the submitted version.
