# Supplementary material for: Cattle-derived knob paratopes grafted onto peripheral loops of the IgG1 Fc region enable the generation of a novel symmetric bispecific antibody format
Source: Front Immunol. 2023 Oct 24;14:1238313. doi: 10.3389/fimmu.2023.1238313 (PMC10628450; doi:10.3389/fimmu.2023.1238313)
Supplement: Supplementary file 1 [file DataSheet_1.docx]

Cattle-derived knob paratopes grafted onto peripheral loops of the IgG1 Fc region enable the generation of a novel symmetric bispecific antibody format

**Desislava Yanakieva^1+^, Lena Vollmer^1+^, Andreas Evers^1^, Vanessa Siegmund^1^, Paul Arras^1^, Lukas Pekar^1^, Achim Doerner^1^, Bernhard Valldorf^3^, Harald Kolmar^2^, Stefan Zielonka^1^, and Simon Krah^1^**

^1^Protein Engineering and Antibody Technologies, Merck Healthcare KGaA, Frankfurter Straße 250, D-64293 Darmstadt, Germany

^2^ Institute for Organic Chemistry and Biochemistry, Technische Universität Darmstadt, Alarich-Weiss-Strasse 4, D-64287 Darmstadt, Germany

*** Correspondence:**

Stefan Zielonka

stefan.zielonka@merckgroup.com 
Simon Krah 
simon.krah@merckgroup.com

^+^ These authors have contributed equally to this work and share first authorship

Supplementary Material

Supplementary Table S1: Heavy chain amino acid sequences the anti-EGFR IgG-based bispecific molecules with engrafted anti-NKp30 bovine knobs.

NKp30 H02 (red) or NKp30 D02 (blue) grafts and linker sequence (green) are highlighted in bold.

| Anti-EGFR IgG (cetuximab) | EVQLVQSGAEVKKPGASVKVSCKASGFSLTNYGVHWMRQAPGQGLEWIGVIWSGGNTDYNTPFTSRVTITSDKSTSTAYMELSSLRSEDTAVYYCARALTYYDYEFAYWGQGTLVTVSSASTKGPSVFPLAPSSKSTSGGTAALGCLVKDYFPEPVTVSWNSGALTSGVHTFPAVLQSSGLYSLSSVVTVPSSSLGTQTYICNVNHKPSNTKVDKRVEPKSCDKTHTCPPCPAPEAAGGPSVFLFPPKPKDTLMISRTPEVTCVVVDVSHEDPEVKFNWYVDGVEVHNAKTKPREEQYNSTYRVVSVLTVLHQDWLNGKEYKCKVSNKALPAPIEKTISKAKGQPREPQVYTLPPSREEMTKNQVSLTCLVKGFYPSDIAVEWESNGQPENNYKTTPPVLDSDGSFFLYSKLTVDKSRWQQGNVFSCSVMHEALHNHYTQKSLSLSPGK |
| --- | --- |
| Anti-EGFR_ EF_H02 | EVQLVQSGAEVKKPGASVKVSCKASGFSLTNYGVHWMRQAPGQGLEWIGVIWSGGNTDYNTPFTSRVTITSDKSTSTAYMELSSLRSEDTAVYYCARALTYYDYEFAYWGQGTLVTVSSASTKGPSVFPLAPSSKSTSGGTAALGCLVKDYFPEPVTVSWNSGALTSGVHTFPAVLQSSGLYSLSSVVTVPSSSLGTQTYICNVNHKPSNTKVDKRVEPKSCDKTHTCPPCPAPEAAGGPSVFLFPPKPKDTLMISRTPEVTCVVVDVSHEDPEVKFNWYVDGVEVHNAKTKPREEQYNSTYRVVSVLTVLHQDWLNGKEYKCKVSNKALPAPIEKTISKAKGQPREPQVYTLPPSREEMTKNQVSLTCLVKGFYPSDIAVEWESNGQPENNYKTTPPVLDSDGSFFLYSKLTV**GGGGSKKSCPEGFSSTRDCPVGSDCLRSDLFARGGNICTWSRQFYATGGGGS**RWQQGNVFSCSVMHEALHNHYTQKSLSLSPGK |
| Anti-EGFR_EF_D02 | EVQLVQSGAEVKKPGASVKVSCKASGFSLTNYGVHWMRQAPGQGLEWIGVIWSGGNTDYNTPFTSRVTITSDKSTSTAYMELSSLRSEDTAVYYCARALTYYDYEFAYWGQGTLVTVSSASTKGPSVFPLAPSSKSTSGGTAALGCLVKDYFPEPVTVSWNSGALTSGVHTFPAVLQSSGLYSLSSVVTVPSSSLGTQTYICNVNHKPSNTKVDKRVEPKSCDKTHTCPPCPAPEAAGGPSVFLFPPKPKDTLMISRTPEVTCVVVDVSHEDPEVKFNWYVDGVEVHNAKTKPREEQYNSTYRVVSVLTVLHQDWLNGKEYKCKVSNKALPAPIEKTISKAKGQPREPQVYTLPPSREEMTKNQVSLTCLVKGFYPSDIAVEWESNGQPENNYKTTPPVLDSDGSFFLYSKLTV**GGGGSRICPDGCGAPTDWDACRALGGGACCMCSSGGGYDFRPVTGGGGS**RWQQGNVFSCSVMHEALHNHYTQKSLSLSPGK |
| Anti-EGFR_AB_H02 | EVQLVQSGAEVKKPGASVKVSCKASGFSLTNYGVHWMRQAPGQGLEWIGVIWSGGNTDYNTPFTSRVTITSDKSTSTAYMELSSLRSEDTAVYYCARALTYYDYEFAYWGQGTLVTVSSASTKGPSVFPLAPSSKSTSGGTAALGCLVKDYFPEPVTVSWNSGALTSGVHTFPAVLQSSGLYSLSSVVTVPSSSLGTQTYICNVNHKPSNTKVDKRVEPKSCDKTHTCPPCPAPEAAGGPSVFLFPPKPKDTLMISRTPEVTCVVVDVSHEDPEVKFNWYVDGVEVHNAKTKPREEQYNSTYRVVSVLTVLHQDWLNGKEYKCKVSNKALPAPIEKTISKAKGQPREPQVYTLPPSR**GGGGSKKSCPEGFSSTRDCPVGSDCLRSDLFARGGNICTWSRQFYATGGGGS**NQVSLTCLVKGFYPSDIAVEWESNGQPENNYKTTPPVLDSDGSFFLYSKLTVDKSRWQQGNVFSCSVMHEALHNHYTQKSLSLSPGK |
| Anti EGFR_AB_D02 | EVQLVQSGAEVKKPGASVKVSCKASGFSLTNYGVHWMRQAPGQGLEWIGVIWSGGNTDYNTPFTSRVTITSDKSTSTAYMELSSLRSEDTAVYYCARALTYYDYEFAYWGQGTLVTVSSASTKGPSVFPLAPSSKSTSGGTAALGCLVKDYFPEPVTVSWNSGALTSGVHTFPAVLQSSGLYSLSSVVTVPSSSLGTQTYICNVNHKPSNTKVDKRVEPKSCDKTHTCPPCPAPEAAGGPSVFLFPPKPKDTLMISRTPEVTCVVVDVSHEDPEVKFNWYVDGVEVHNAKTKPREEQYNSTYRVVSVLTVLHQDWLNGKEYKCKVSNKALPAPIEKTISKAKGQPREPQVYTLPPSR**GGGGSRICPDGCGAPTDWDACRALGGGACCMCSSGGGYDFRPVTGGGGS**NQVSLTCLVKGFYPSDIAVEWESNGQPENNYKTTPPVLDSDGSFFLYSKLTVDKSRWQQGNVFSCSVMHEALHNHYTQKSLSLSPGK |

Supplementary Table S2: Heavy chain amino acid sequences the anti-NKp46 IgG-based bispecific molecules with engrafted anti-EGFR bovine knobs.

EGFR-H05 (orange) or EGFR-F06 (purple) knob domains and linker sequence (green) are highlighted in bold.

| Anti-NKp46 IgG | QVQLVQSGAEVKKPGSSVKVSCKASGYTFSDYVINWVRQAPGQGLEWMGEIYPGSGTNYYNEKFKAKATITADKSTSTAYMELSSLRSEDTAVYYCARRGRYGLYAMDYWGQGTTVTVSSASTKGPSVFPLAPSSKSTSGGTAALGCLVKDYFPEPVTVSWNSGALTSGVHTFPAVLQSSGLYSLSSVVTVPSSSLGTQTYICNVNHKPSNTKVDKRVEPKSCDKTHTCPPCPAPEAAGGPSVFLFPPKPKDTLMISRTPEVTCVVVDVSHEDPEVKFNWYVDGVEVHNAKTKPREEQYNSTYRVVSVLTVLHQDWLNGKEYKCKVSNKALPAPIEKTISKAKGQPREPQVYTLPPSREEMTKNQVSLTCLVKGFYPSDIAVEWESNGQPENNYKTTPPVLDSDGSFFLYSKLTVDKSRWQQGNVFSCSVMHEALHNHYTQKSLSLSPGK |
| --- | --- |
| Anti-NKp46_EF_H05 | QVQLVQSGAEVKKPGSSVKVSCKASGYTFSDYVINWVRQAPGQGLEWMGEIYPGSGTNYYNEKFKAKATITADKSTSTAYMELSSLRSEDTAVYYCARRGRYGLYAMDYWGQGTTVTVSSASTKGPSVFPLAPSSKSTSGGTAALGCLVKDYFPEPVTVSWNSGALTSGVHTFPAVLQSSGLYSLSSVVTVPSSSLGTQTYICNVNHKPSNTKVDKRVEPKSCDKTHTCPPCPAPEAAGGPSVFLFPPKPKDTLMISRTPEVTCVVVDVSHEDPEVKFNWYVDGVEVHNAKTKPREEQYNSTYRVVSVLTVLHQDWLNGKEYKCKVSNKALPAPIEKTISKAKGQPREPQVYTLPPSREEMTKNQVSLTCLVKGFYPSDIAVEWESNGQPENNYKTTPPVLDSDGSFFLYSKLTV**GGGGSKLKSCPDGYTSGVECRFRGYTCANDGCWRVCSFTTCSGWMPASDTGGGGS**RWQQGNVFSCSVMHEALHNHYTQKSLSLSPG |
| Anti-NKp46_EF_F06 | QVQLVQSGAEVKKPGSSVKVSCKASGYTFSDYVINWVRQAPGQGLEWMGEIYPGSGTNYYNEKFKAKATITADKSTSTAYMELSSLRSEDTAVYYCARRGRYGLYAMDYWGQGTTVTVSSASTKGPSVFPLAPSSKSTSGGTAALGCLVKDYFPEPVTVSWNSGALTSGVHTFPAVLQSSGLYSLSSVVTVPSSSLGTQTYICNVNHKPSNTKVDKRVEPKSCDKTHTCPPCPAPEAAGGPSVFLFPPKPKDTLMISRTPEVTCVVVDVSHEDPEVKFNWYVDGVEVHNAKTKPREEQYNSTYRVVSVLTVLHQDWLNGKEYKCKVSNKALPAPIEKTISKAKGQPREPQVYTLPPSREEMTKNQVSLTCLVKGFYPSDIAVEWESNGQPENNYKTTPPVLDSDGSFFLYSKLTV**GGGGSAKCPDGYSDARHCRDGCDCFGWDCFRSGAWAECYYEVYTGGGGS**RWQQGNVFSCSVMHEALHNHYTQKSLSLSPGK |
| Anti-NKp46_AB_H05 | QVQLVQSGAEVKKPGSSVKVSCKASGYTFSDYVINWVRQAPGQGLEWMGEIYPGSGTNYYNEKFKAKATITADKSTSTAYMELSSLRSEDTAVYYCARRGRYGLYAMDYWGQGTTVTVSSASTKGPSVFPLAPSSKSTSGGTAALGCLVKDYFPEPVTVSWNSGALTSGVHTFPAVLQSSGLYSLSSVVTVPSSSLGTQTYICNVNHKPSNTKVDKRVEPKSCDKTHTCPPCPAPEAAGGPSVFLFPPKPKDTLMISRTPEVTCVVVDVSHEDPEVKFNWYVDGVEVHNAKTKPREEQYNSTYRVVSVLTVLHQDWLNGKEYKCKVSNKALPAPIEKTISKAKGQPREPQVYTLPPSR**GGGGSKLKSCPDGYTSGVECRFRGYTCANDGCWRVCSFTTCSGWMPASDTGGGGS**NQVSLTCLVKGFYPSDIAVEWESNGQPENNYKTTPPVLDSDGSFFLYSKLTVDKSRWQQGNVFSCSVMHEALHNHYTQKSLSLSPGK |
| Anti-NKp46_AB_F06 | QVQLVQSGAEVKKPGSSVKVSCKASGYTFSDYVINWVRQAPGQGLEWMGEIYPGSGTNYYNEKFKAKATITADKSTSTAYMELSSLRSEDTAVYYCARRGRYGLYAMDYWGQGTTVTVSSASTKGPSVFPLAPSSKSTSGGTAALGCLVKDYFPEPVTVSWNSGALTSGVHTFPAVLQSSGLYSLSSVVTVPSSSLGTQTYICNVNHKPSNTKVDKRVEPKSCDKTHTCPPCPAPEAAGGPSVFLFPPKPKDTLMISRTPEVTCVVVDVSHEDPEVKFNWYVDGVEVHNAKTKPREEQYNSTYRVVSVLTVLHQDWLNGKEYKCKVSNKALPAPIEKTISKAKGQPREPQVYTLPPSR**GGGGSAKCPDGYSDARHCRDGCDCFGWDCFRSGAWAECYYEVYTGGGGS**NQVSLTCLVKGFYPSDIAVEWESNGQPENNYKTTPPVLDSDGSFFLYSKLTVDKSRWQQGNVFSCSVMHEALHNHYTQKSLSLSPGK |


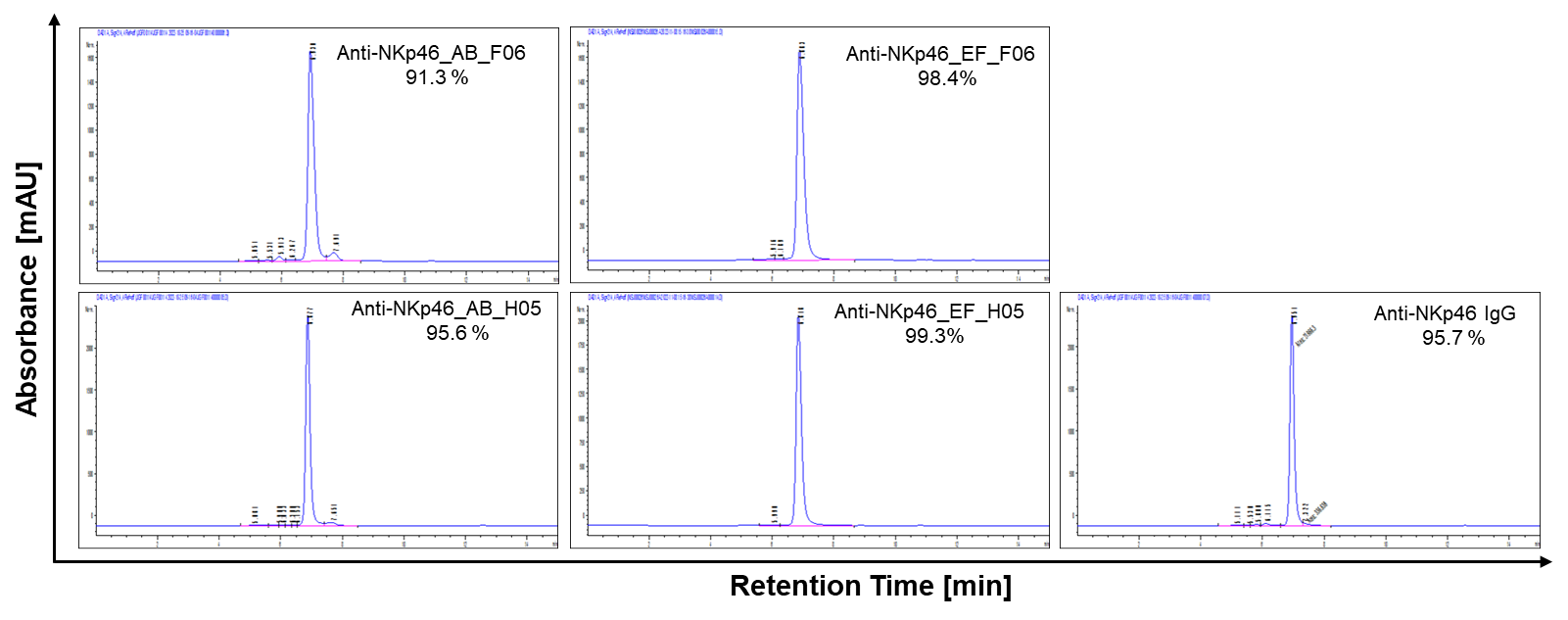


Supplementary Figure S1: Exemplary size exclusion high performance liquid chromatography (SE-HPLC) data of bispecific anti-NKp46×knob molecules in comparison to parental monospecific anti-NKp46 IgG


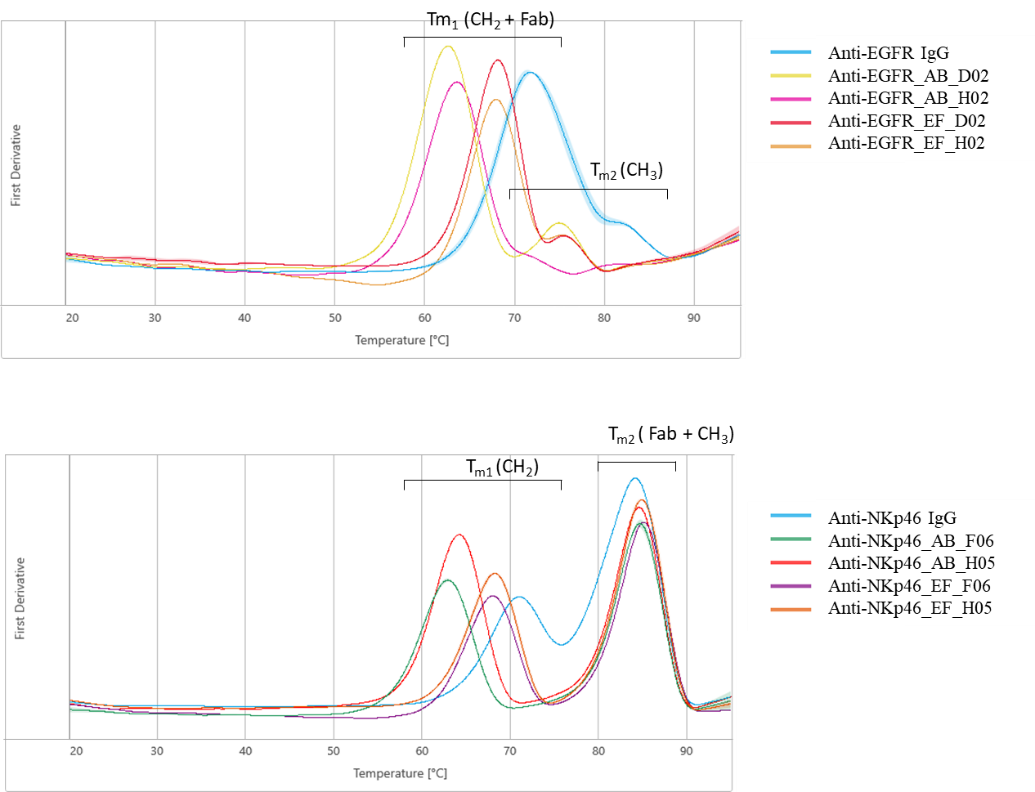


**Supplementary Figure S2:** Thermal unfolding of CH3 loop-grafted antibodies by differential scanning fluorimetry. Overlays of the melting curves for AB and EF loop-grafted antibodies compared to the respective IgG Fc antibody control were recorded utilizing a temperature gradient from 20 °C to 95 °C at a slope of 1 °C/min. First derivatives of 350 nm / 330 nm curves are shown.


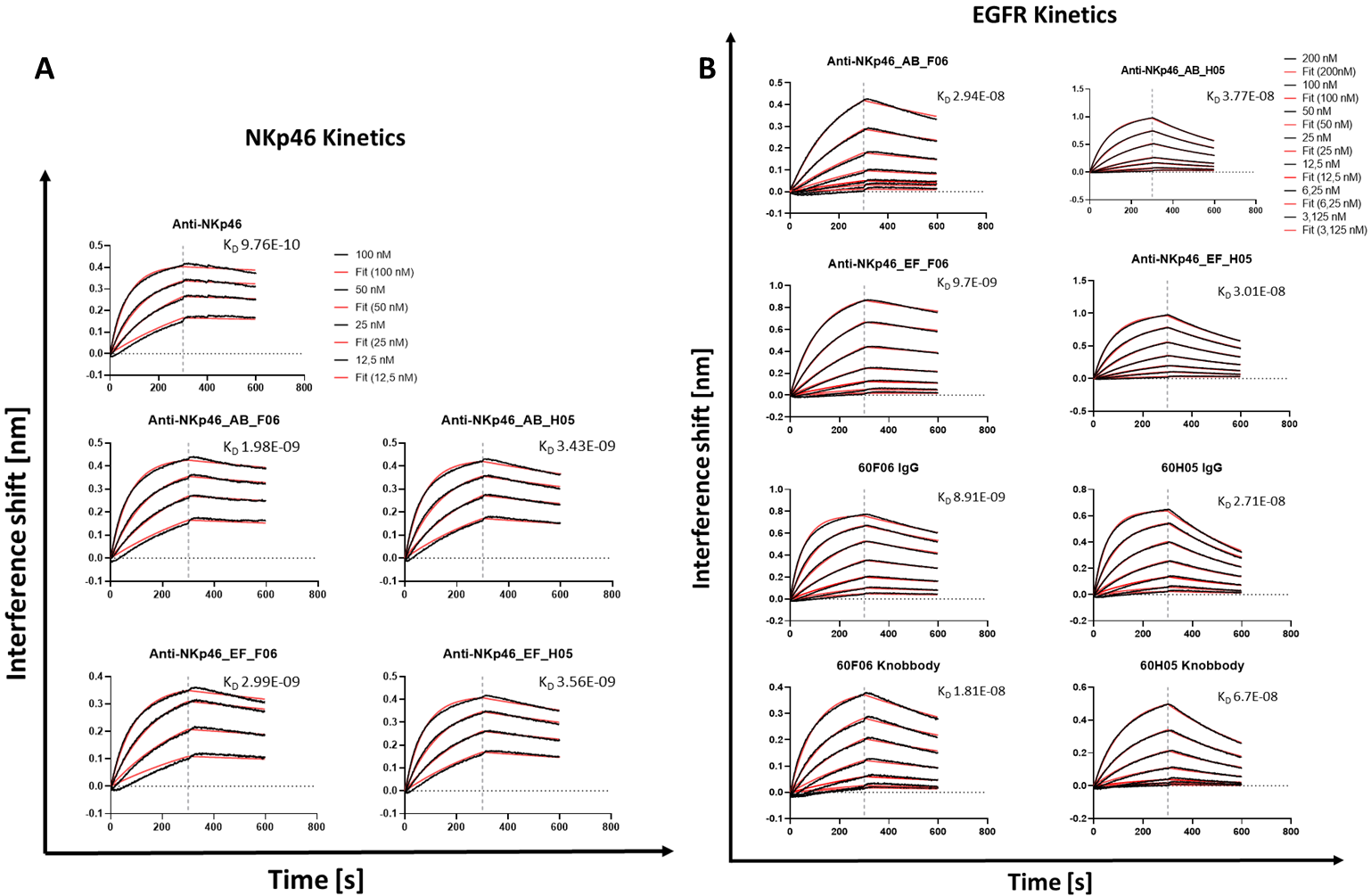


Supplementary Figure S3: (A) Kinetic measurements of bispecific NKp46xEGFR-targeting constructs against NKp46.

5 µg/mL of antibodies were loaded on AHC tips. After 60 sec of sensor rinsing, the antigen binding was conducted between 100 and 1.6 nM (1:2 serial dilution) for 300 sec. Following the association, dissociation of the antigen was conducted for 300 sec in KB. For K_D_ determination interference shifts were fitted at concentrations between 100 and 12.5 nM. (B) Kinetic measurements of bispecific NKp46xEGFR-targeting constructs against EGFR. 5 µg/mL of antibodies were loaded on FAB2G (except chimeric cattle IgG and Knobbody constructs which were loaded on AHC tips). After 60 sec of sensor rinsing, the antigen binding was conducted between 200 and 3.12 nM (1:2 serial dilution) for 300 sec. Following the association, dissociation of the antigen was conducted for 300 sec in KB. For K_D_ determination interference shifts were fitted at concentrations between 200 and 3.12 nM.


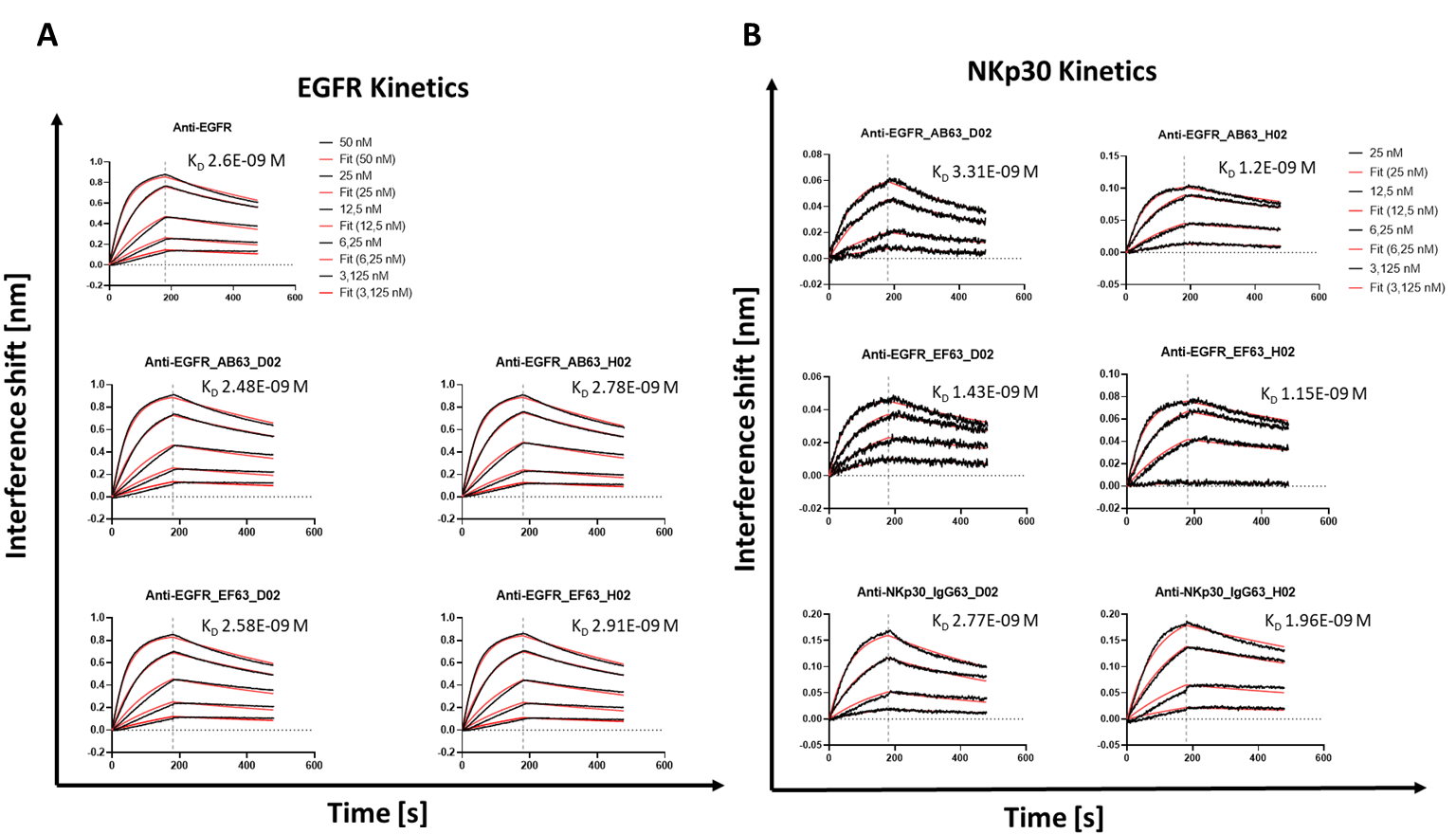


Supplementary Figure S4: (A) Kinetic measurements of bispecific EGFRxNKp30-targeting constructs against EGFR.

5 µg/mL antibodies were loaded on AHC tips. After 60 sec of sensor rinsing, the antigen binding was conducted at between 200 and 3.12 nM (1:2 serial dilution) for 180 secs. Following the association, dissociation of the antigen was conducted for 300 sec in KB. For K_D_ determination interference shifts were fitted, at concentrations between 50 and 3.125 nM. (B) Kinetic measurements of bispecific EGFRxNKp30-targeting constructs against NKp30.

5 µg/mL antibodies were loaded on FAB2G tips (except cow-IgG were loaded on AHC tips). After 60 sec of sensor rinsing, the antigen binding was conducted between 100 and 1.6 nM (1:2 serial dilution) for 180 sec. Following the association, dissociation of the antigen was conducted for 300 sec in KB. For K_D_ determination interference shifts were fitted at concentrations between 25 and 3.12 nM.

Figure S5: Time-resolved NK cell cytotoxicity mediated by the bispecific NKp46×EGFR-targeting constructs.

Fluorescence-microscopy based NK cell-mediated killing assay using EGFR-positive A431 target cells and PBMC-derived NK effector cells at an E:T ratio of 5:1. Analysis target cell killing kinetics at 10 nM sample concentration. All values were normalized to maximum killing of cetuximab at 10 nM after 24 hours. Basal killing was not subtracted and is shown as a separate graph (brown). Graphs show normalized means ± SEM of n=4 different healthy donors.


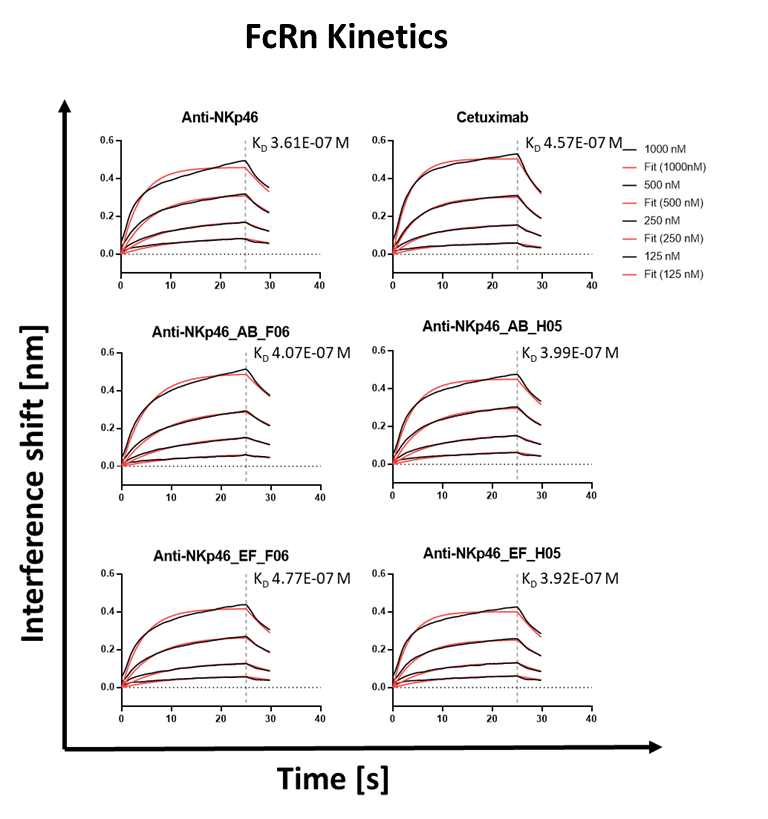


Supplementary Figure S6: Kinetic measurements of NKp46xEGFR-targeting constructs against human FcRn.

5 µg/mL antibodies were loaded on FAB2G tips. For efficient binding of FcRn to IgGs under acidic conditions, PBS at pH 6.0 + 0.05% Tween for dilution of analyte samples as well as baseline and dissociation assay steps was used. After 60 sec of sensor rinsing, the antigen binding was conducted between 1000 and 31.25 nM (1:2 serial dilution) for 25 sec. Following the association, dissociation of the antigen at pH 6 was conducted for 5 sec. For K_D_ determination interference shifts were fitted at concentrations between 1000 and 125 nM.


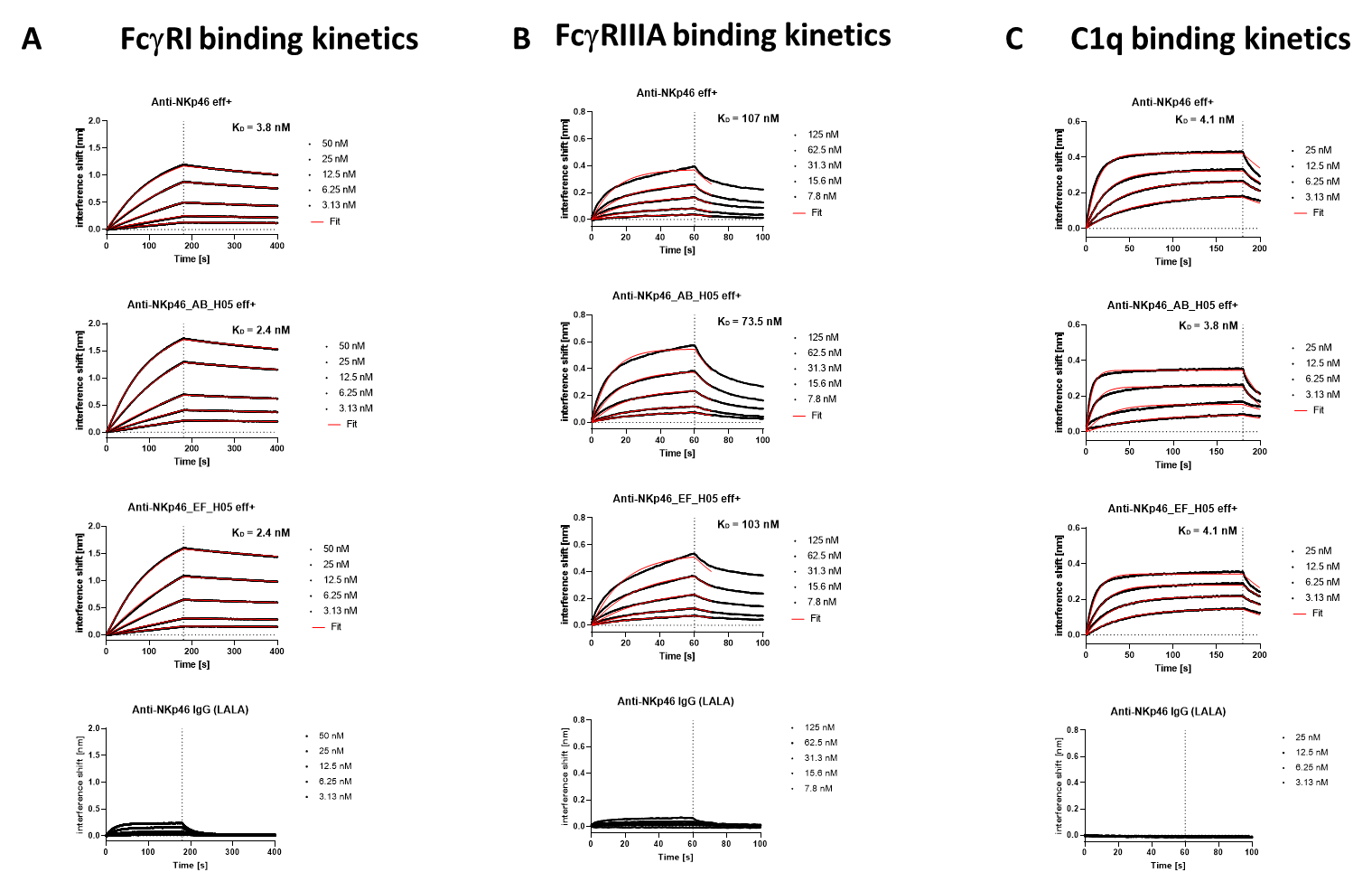


**Figure S7:** Assessment of Fc effector functioning after engraftment of bovine-derived knobs into the CH3 domain. (A) Binding kinetics of high-affinity FcγRI (CD64) receptor. FcγRI-His6 (5 µg/mL) was loaded on HIS1K biosensors for 180 s. After 60 sec of sensor rinsing, antibody binding was conducted at concentrations between 50 and 1.56 nM (1:2 serial dilution) for 180 secs. Antibody dissociation was conducted for 600 sec in KB. For K_D_ determination interference shifts were fitted, at concentrations between 50 and 3.125 nM. (B) Binding kinetics of FcgRIIIA (CD16a) receptor. FcgRIIIA-His6 (5 µg/mL) was loaded on HIS1K biosensors for 180 s. After 60 sec of sensor rinsing, antibody binding was conducted at concentrations between 250 and 3.9 nM (1:2 serial dilution) for 60 secs. Antibody dissociation was conducted for 60 sec in KB. For K_D_ determination interference shifts were fitted, at concentrations between 125 and 7.8 nM and the first 10 s dissociation were used. (C) Binding kinetics of C1q complex. Antibodies were immobilized on ProteinL for biosensors at concentration of 5 µg/mL for 180 s. Following a 60 sec sensor rinsing step, active C1q complex was associated for 180 s at concentrations between 100 nM and 1.56 nM. C1q dissociation was conducted for 60 s. For K_D_ determination interference shifts were fitted, at concentrations between 25 and 3.13 nM and the first 10 s dissociation were used.

**Figure S8:** Complement-dependent cytotoxicity (CDC) assay using A431 cells (EGFR-positive) and baby rabbit complement. Target cells were treated with 10 nM effector-positive antibody constructs or a combination of cetuximab wt and effector-positive IgGxknob bispecific. Data shows mean values of two independent experiments performed in biological triplicates. One-way ANOVA statistical analysis for data significance in comparison to complement only (negative control) was performed and indicated.
